# *Streptococcus salivarius* Probiotics to Prevent Acute Otitis Media in Children: A Randomized Clinical Trial

**DOI:** 10.1001/jamanetworkopen.2023.40608

**Published:** 2023-11-02

**Authors:** Suvi Sarlin, Ulla Koskela, Minna Honkila, Paula A. Tähtinen, Tytti Pokka, Marjo Renko, Terhi Tapiainen

**Affiliations:** 1 Department of Pediatrics and Adolescent Medicine and Medical Research Center, Oulu University Hospital, Oulu, Finland; 2 Research Unit of Clinical Medicine, University of Oulu, Oulu, Finland; 3 Department of Paediatrics and Adolescent Medicine, Turku University Hospital and University of Turku, Turku, Finland; 4 Research Service Unit, Oulu University Hospital, Oulu, Finland; 5 Department of Pediatrics, Kuopio University Hospital and University of Eastern Finland, Kuopio, Finland

## Abstract

**Importance:**

New approaches for the prevention of acute otitis media (AOM), the most common reason for antibiotic use in children, are needed.

**Objective:**

To assess the efficacy of the *Streptococcus salivarius* K12 oral probiotics in the primary prevention of AOM.

**Design, Setting, and Participants:**

This double-blind, randomized placebo-controlled clinical trial was conducted from August 1, 2020, to May 31, 2021, at 50 day care centers in the Oulu region of Finland. A total of 827 children aged 1 to 6 years attending day care were included. The exclusion criteria consisted of ongoing antimicrobial prophylaxis or immunodeficiency. The follow-up time was 6 months and was completed on May 31, 2021. Data were analyzed from October 24, 2022, to September 16, 2023, based on intention to treat.

**Intervention:**

Eligible participants were randomly allocated to receive 1 daily dose of a *S salivarius* K12 product or placebo every evening for 6 months. A daily dose was defined as 1 sachet of soluble oral powder for children younger than 3 years or 1 chewable tablet for children 3 years or older containing 1 × 10^9^ colony-forming units of *S salivarius* K12.

**Main Outcomes and Measures:**

The primary outcome was the proportion of children with at least 1 episode of AOM requiring antimicrobial therapy within 6 months of randomization. All physician visits and purchases of antimicrobial drugs were retrieved from the electronic national medical record and prescription register. The primary outcome was met if the legal guardian had purchased an antimicrobial prescription for AOM.

**Results:**

A total of 827 children with a mean (SD) age of 4.1 (1.6) years (433 boys [52.4%]) were randomized to *S salivarius* K12 oral products (n = 413) or placebo (n = 414). Thirty-four children (8.2%) in the *S salivarius* group and 24 children (5.8%) in the placebo group experienced at least 1 episode of AOM requiring antimicrobial therapy during the 6-month follow-up period (relative risk, 1.42 [95% CI, 0.86-2.34]; proportion difference, −2.44% [95% CI, −5.94% to 1.09%]; *P* = .17). Time to first AOM episode did not differ between the groups (174 [95% CI, 171-177] days in the *S salivarius* group vs 176 [95% CI, 173-179] days in the placebo group; *P* = .18).

**Conclusions and Relevance:**

In this randomized placebo-controlled clinical trial, the daily use of the *S salivarius* K12 products for 6 months did not reduce the occurrence of AOM. New approaches for primary prevention of AOM among children are needed.

**Trial Registration:**

ClinicalTrialsRegister.eu Identifier: 2020-001076-14

## Key Points

**Question:**

Are *Streptococcus salivarius* K12 oral probiotic products effective in the primary prevention of acute otitis media (AOM) among children attending day care?

**Findings:**

In this randomized clinical trial of 827 children aged 1 to 6 years, the daily use of a *S salivarius* K12 oral probiotic product for 6 months did not reduce the occurrence of AOM.

**Meaning:**

These findings suggest that oral *S salivarius* K12 should not be recommended for prevention of AOM in children.

## Teaser

This randomized clinical trial investigates the clinical effectiveness of commercially available *Streptococcus salivarius* K12 oral probiotic products in the primary prevention of acute otitis media in children attending day care.

## Introduction

Acute otitis media (AOM) is an ascending infection associated with Eustachian tube dysfunction during respiratory viral infection, allowing otopathogens to ascend from the nasopharynx to the middle ear. Prevention of AOM plays a significant role in reducing the overall use of antibiotics in children.^1^ Pneumococcal conjugate vaccines (PCVs) have been shown to reduce the occurrence of AOM, with estimates ranging from 8% to 10% for 7-valent PCV^2,3^ and up to 23% for 10-valent PCV.^4,5^ Influenza vaccines also reduce the occurrence of AOM during influenza epidemics.^6,7^ Otherwise, the current options for primary prevention of AOM are limited.

Lactobacilli, which mainly belong to the microbiome of the gastrointestinal tract, only marginally colonize the nasopharyngeal microbiome and have largely proven ineffective in preventing AOM.^8,9,10^ A previous randomized clinical trial (RCT)^11^ found that an in-house α-streptococcal spray administered nasally after antimicrobial therapy for AOM protected children from recurrent AOM. However, in-house bacterial sprays have not been evaluated for safety and may vary in effectiveness. Recently, commercially available probiotic products containing *Streptococcus salivarius* strains, successful colonizers of the human oral cavity and nasopharynx,^12,13^ have been developed for oral health care.^14,15^ Sarlin et al^13^ have previously shown in an RCT that commercially available *S salivarius* K12 oral products may reduce the relative abundance of otopathogens in the nasopharyngeal microbiome while maintaining its diversity. We hypothesized that *S salivarius* K12 oral products may be effective in preventing AOM. In this placebo-controlled RCT, we investigated the clinical efficacy of commercially available streptococcal strain *S salivarius* K12 oral probiotic products in the primary prevention of AOM among children attending day care.

## Methods

The Finnish Medical Agency and the Ethical Committee of the North Ostrobothnia’s Hospital District, Oulu, Finland, approved the trial protocol. Children whose legal guardians provided written informed consent were included in the trial. The trial followed the Consolidated Standards of Reporting Trials (CONSORT) reporting guideline. The study protocol and statistical analysis plan are provided in Supplement 1.

### Trial Design

This RCT was conducted in 50 day care centers in the Oulu region of Finland and in the Department of Pediatrics and Adolescent Medicine, Oulu University Hospital, Finland, from August 1, 2020, to May 31, 2021. Participants were recruited between August 26 and November 25, 2020. Six-month follow-up was completed on May 31, 2021. Throughout the study period, during the COVID-19 pandemic, all day care centers and primary schools remained open in Finland.

### Participants

Eligible participants were children aged 1 to 6 years attending day care in the area. The exclusion criteria were ongoing antimicrobial prophylaxis or any immunodeficiency. The use of other probiotic products was discouraged but was not an exclusion criterion. Finnish language skill was an inclusion criterion. The families did not receive any compensation for participating in the trial.

### Randomization and Blinding

Eligible participants were randomly assigned at a 1:1 ratio to receive 1 daily dose of an *S salivarius* K12 oral product or placebo every evening after toothbrushing for 6 months. Randomization was stratified according to children’s age (<3 years for oral powder and ≥3 years for chewable tablets). A biostatistician created a randomization list with computerized block randomization using permuted blocks in variable sizes.

Before the study, all identical *S salivarius–*containing and placebo-containing study packages were individually numbered according to a randomization list created by the biostatistician (T.P.). Randomization and numbering was completed by personnel not participating in the recruitment or follow-up of the patients. After the legal guardian signed the informed consent form, the participant was given the next free study number and the product assigned to it at the day care center.

The participating children, their guardians, and the study physicians were blinded to the assignment of the *S salivarius* K12 and placebo groups. The products were packed in similar packages, and there were no labels on them, except the randomization number. Both *S salivarius* and placebo products looked and tasted the same.

### Interventions

The families gave the study products to the children according to the instructions. A quantity of 1 × 10^9^ colony forming units (CFUs) of *S salivarius* K12 administered once daily has previously been shown to successfully colonize the nasopharynx in adults.^8,16^ A daily dose was defined as 1 sachet of soluble oral powder for children younger than 3 years and 1 chewable tablet for children 3 years or older. One sachet of soluble oral powder contained 1 × 10^9^ CFUs of *S salivarius* K12 with 1010 mg of maltodextrin (bulking agent), 480 mg of fructo-oligosaccharide (bulking agent), and 10 mg of strawberry flavor. One oral chewable tablet contained 1 × 10^9^ CFUs of *S salivarius* K12 with 789 mg of isomaltitol, 72.2 mg of xylitol, 6.9 mg of silicon dioxide, 10 mg of peppermint flavor, 400 IU of vitamin D_3_, 1 × 10^8^ CFUs of *Lactobacillus rhamnosus* GG, and 1 × 10^8^ CFUs of *Propionibacterium shermanii*. The placebo soluble oral powder contained 1010 mg of maltodextrin, 480 mg of fructo-oligosaccharide, and 10 mg of strawberry flavor. The placebo oral chewable tablet contained 789 mg of isomaltitol, 72.2 mg of xylitol, 6.9 mg of silicon dioxide, 10 mg of peppermint flavor, and 400 IU of vitamin D_3_. All study products were purchased from GutGuide Ltd.

### Recruitment and Clinical Follow-Up

Study physicians visited day care centers to provide families with information about the study. In addition, local child health clinics distributed study information brochures to families. Participants with written informed consent from a legal guardian were included in the study. After this, the participant was given the next available study number and the randomized study product adjoined to it. After enrollment, families were sent an online questionnaire about background characteristics. In Finland, social security number is binomial according to sex. Participants’ sex was calculated from social security number. No ethnicity data was collected.

Study physicians reviewed all purchases of antimicrobials from the comprehensive electronic national prescription register during the 6 months of follow-up.^17^ The children’s medical records from the national electronic medical record register^17^ were reviewed. Study physicians (S.S. and U.K.) recorded whether the indication for the antimicrobial prescription had been AOM, defined in accordance with the national guidelines.^18^ In addition, monthly online questionnaires were sent to families.

The diagnosis of AOM was defined according to the national AOM treatment guideline as an acute, short-term, and clinically diagnosed episode of otitis media, with the presence of middle-ear effusion, signs of inflammation of the tympanic membrane, and at least 1 sign or symptom of an acute respiratory infection.^18^ Recurrent AOM was defined as at least 3 episodes of AOM within 6 months. To improve the accuracy of AOM diagnosis in outpatient care, families were sent an electronic AOM diagnostic tool with 8 images of the middle ear, including purulent AOM (Supplement 1). If the children visited their own physicians for suspected AOM, the families were advised to present the images.

### Outcomes

The primary outcome was the proportion of children with at least 1 episode of AOM requiring antimicrobial therapy confirmed from the electronic national medical record and prescription register^17^ within 6 months of randomization. The primary outcome was met if the guardian had purchased an antimicrobial prescription for the child within 6 months of randomization for an AOM diagnosis defined according to national guidelines.^18^

The secondary outcomes retrieved from the electronic national medical record and prescription register^17^ were time to the first episode of AOM requiring antimicrobial therapy, the proportion of children with recurrent AOM requiring antimicrobial therapy, the proportion of children receiving any antimicrobial therapy, the proportion of children with any physician’s appointment due to acute illness, the proportion of children hospitalized for an acute respiratory illness, and the duration of hospitalization. The secondary outcomes from questionnaire data were other physician-diagnosed AOM episodes, the number of days with respiratory or gastroenteritis symptom, and the number of days of parental absence from work due to their child’s illness per person-years at risk (PYR). All secondary outcomes were reported within 6 months of randomization.

Exploratory post hoc analyses, added to the statistical analysis plan after study initiation but before completion of other data analyses, were conducted for the proportion of children with AOM with a complication, the number of AOM episodes with a complication, the proportion of children with tympanostomy tube insertion or adenoidectomy, the proportion of children with respiratory symptoms leading to SARS-CoV-2 testing, the proportion of children with a COVID-19 infection or a positive SARS-CoV-2 test result, and the number of days of a child’s absence from day care per PYR. All exploratory outcomes were reported within 6 months of randomization.

### Sample Size

Due to social distancing measures during the COVID-19 pandemic, the occurrence of AOM decreased. We therefore recalculated the sample size during the study to ensure statistical power. We assumed that the proportion of children in the control group with at least 1 episode of AOM would decrease from 30% to 12% within 6 months because pediatric emergency department visits were reduced by approximately 60% in Finland during the COVID-19 pandemic.^19^ We considered efficacy to be clinically meaningful if the occurrence of AOM was further decreased by 50% in the intervention group (ie, the proportion of children with at least 1 episode of AOM decreased from 12% to 6% within 6 months). With a statistical power of 80% and a 2-sided α error of 5%, the sample size needed was 389 children per group.

### Statistical Analysis

Statistical analyses were performed according to the intention-to-treat principle from October 24, 2022, to September 16, 2023. We calculated the 95% CIs of the differences between groups using a standard normal deviate test for the proportions,^20^ as well as a 2-tailed unpaired *t* test for Gaussian continuous variables; *P* < .05 was considered statistically significant. Relative risks with 95% CIs were calculated. A Mann-Whitney test was performed for non-Gaussian variables. Time to first episode of AOM requiring antimicrobial therapy during the intervention within 6 months was analyzed with the Kaplan-Meier method, and the log-rank test was used to compare survival distributions. The data were analyzed with SPSS Statistics software for Windows, version 27.0 (IBM Corporation), and StatsDirect statistical software, version 3.3.5 (StatsDirect Ltd).

## Results

**Figure 1.**
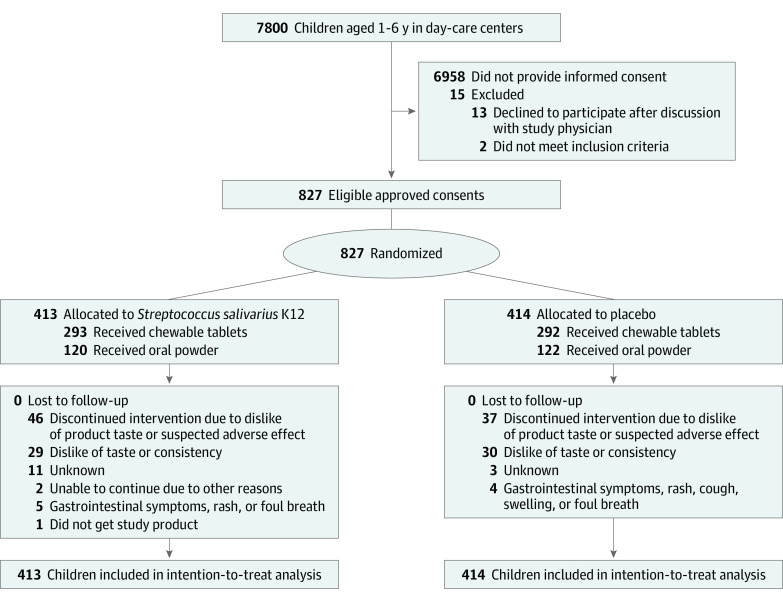
Study Flow Diagram

Altogether, 827 children were included in the intention-to-treat analyses (mean [SD] age, 4.1 [1.6] years; 394 girls [47.6%] and 433 boys [52.4%]), with 413 children in the *S salivarius* group and 414 in the placebo group (Figure 1). The mean (SD) age of the children was similar in both groups (Table 1). The mean (SD) duration of any breastfeeding was 5.0 (4.0) months in the *S salivarius* group and 5.2 (4.6) months in the placebo group. A total of 77 of 380 parents (20.3%) in the *S salivarius* group and 68 of 388 (17.5%) in the placebo group with data available reported parental smoking. Tympanostomy tubes had been inserted for 64 of 380 children (16.8%) in the *S salivarius* group and 57 of 388 (14.7%) in the placebo group with data available before study entry. In total, 46 children (11.1%) in the *S salivarius* group and 37 (8.9%) in the placebo group stopped using the study product earlier than planned.

**Table 1.** Baseline Characteristics of the Study Participants^a^

Characteristic	Study group	
	*Streptococcus salivarius* K12 intervention (n = 413)	Placebo (n = 414)
Age, mean (SD), y	4.1 (1.6)	4.1 (1.6)
Sex		
Female	194 (47.0)	200 (48.3)
Male	219 (53.0)	214 (51.7)
Cystic fibrosis	0	0
Primary ciliary disease	0	0
10-Valent PCV vaccination^b^	368 (96.8)	375 (96.6)
Influenza vaccination^c^	123 (45.6)	133 (48.7)
Any allergy^b^	53 (13.9)	71 (18.3)
Underlying illness requiring ongoing medication^b^	15 (3.9)	21 (5.4)
Current use of other probiotic products^b^^,^^d^	59 (15.5)	59 (15.2)
Any antimicrobial therapy during the preceding 6 mo before study entry^b^	43 (11.3)	47 (12.1)
Risk factors for AOM^b^		
No. of siblings		
0	68 (17.9)	64 (16.5)
1	170 (44.7)	183 (47.2)
≥2	142 (37.4)	141 (36.3)
Parental smoking	77 (20.3)	68 (17.5)
Current pacifier use	20 (5.3)	22 (5.7)
Former pacifier use	262 (68.9)	277 (71.4)
Duration of breastfeeding, mean (SD), mo	5.0 (4.0)	5.2 (4.6)
AOM-related surgery^b^		
Tympanostomy	64 (16.8)	57 (14.7)
Presence of tympanostomy tubes	22 (5.8)	23 (5.9)
Adenoidectomy	24 (6.3)	25 (6.4)

Abbreviations: AOM, acute otitis media; PCV, pneumococcal conjugate vaccine.

^a^ Unless otherwise indicated, data are expressed as No. (%) of 413 in the *S salivarius* K12 and 414 children in the placebo group.

^b^ Data are available from 380 children in the *Streptococcus salivarius* K12 group and 388 in the placebo group.

^c^ Data are available from 270 children in the *Streptococcus salivarius* K12 group and 273 in the placebo group.

^d^ Includes Lactobacilli or *Saccharomyces* species.

### Primary Outcome

In total, 34 children (8.2%) in the *S salivarius* group and 24 (5.8%) in the placebo group had at least 1 episode of AOM requiring antimicrobial therapy during the 6-month follow-up period. The difference between the 2 groups was not statistically significant (relative risk, 1.42 [95% CI, 0.86-2.34]; proportion difference, −2.44% [95% CI −5.94% to 1.09%]; *P* = .17) (Table 2).

**Table 2.** Primary and Secondary Outcomes in *S salivarius* K12 Group vs Placebo Group

Outcome	Study group		Proportion or mean difference (95% CI)	Relative risk (95% CI)	*P* value
	Placebo (n = 414)	*Streptococcus salivarius* K12 (n = 413)			
**Primary **					
National prescription register and medical record data, No. (%)					
≥1 Episode of AOM requiring antimicrobial therapy, No. (%)	24 (5.8)	34 (8.2)	−2.44 (−5.94 to 1.09)	1.42 (0.86 to 2.34)	.17
Oral powder (122 in placebo and 120 in *S salivarius* K12 groups)	11 (9.0)	24 (20.0)	−10.98 (−20.11 to −2.17)	2.22 (1.16 to 4.30)	.02^a^
Chewable tablet (292 in placebo and 293 in *S. salivarius* K12 groups)	13 (4.5)	10 (3.4)	1.04 (−2.26 to 4.46)	0.77 (0.35 to 1.68)	.52
**Secondary **					
National prescription register and medical record data					
Recurrent otitis media, No. (%)					
≥2 AOM episodes in 6 mo	6 (1.4)	9 (2.2)	−0.73 (−2.81 to 1.22)	1.50 (0.56 to 4.02)	.33
≥3 AOM episodes in 6 mo	2 (0.5)	4 (1.0)	−0.49 (−1.81 to 0.84)	2.00 (0.43 to 9.32)	.41
Any antimicrobial therapy, No. (%)	37 (8.9)	49 (11.9)	−2.93 (−7.09 to 1.27)	1.33 (0.89 to 1.99)	.17
≥1 Physician’s appointment due to acute illness, No. (%)	93 (22.5)	85 (20.6)	1.88 (−3.73 to 7.47)	0.92 (0.71 to 1.19)	.51
Telephone or video appointment, No. (%)	32 (7.7)	47 (11.4)	−3.65 (−7.66 to 0.39)	1.47 (0.96 to 2.25)	.07
Hospitalized for an acute respiratory illness, No. (%)	0	4 (1.0)	−0.97 (−2.11 to 0.19)	0 (0 to 0.97)	.05
Duration of hospitalization, median (IQR), d	0 (0.00-0.00)	3 (2.00 to 3.00)	NA	NA	NA
Questionnaire data^b^					
No. of AOM episodes per PYR, median (IQR)	0.00 (0.00 to 0.00)	0.00 (0.00 to 0.00)	−0.14 (−0.34 to 0.07)	NA	.16
No. of days with respiratory or gastroenteritis symptoms per PYR, median (IQR)					
Runny nose	21.29 (7.60 to 40.96)	4.06 (6.08 to 44.61)	−1.5 (−7.13 to 4.06)	NA	.97
Cough	8.11 (0.00 to 29.30)	8.11 (0.00 to 26.36)	1.10 (−3.88 to 6.15)	NA	.62
Sore throat	0.00 (0.00 to 4.06)	0.00 (0.00 to 4.06)	0.12 (−1.57 to 1.80)	NA	.85
Gastrointestinal tract discomfort or pain	0.00 (0.00 to 2.03)	0.00 (0.00 to 2.03)	0.36 (−1.63 to 2.35)	NA	.42
Fever	0.00 (0.00 to 0.00)	0.00 (0.00 to 0.00)	−0.30 (−1.14 to 0.53)	NA	.60
Earache	0.00 (0.00 to 0.00)	0.00 (0.00 to 0.00)	−0.87 (−2.18 to 0.44)	NA	.20
Diarrhea	0.00 (0.00 to 0.00)	0.00 (0.00 to 0.00)	0.36 (−0.99 to 1.71)	NA	.77
Wheezing	0.00 (0.00 to 0.00)	0.00 (0.00 to 0.00)	−0.08 (−0.78 to 0.62)	NA	.83
Vomiting	0.00 (0.00 to 0.00)	0.00 (0.00 to 0.00)	0.05 (−0.23 to 0.33)	NA	.41
No. of days of parental absence from work due to the child’s acute respiratory illness per PYR, mean (SD)	6.08 (0.00 to 20.28)	4.06 (0.00 to 16.22)	−0.56 (−2.08 to 3.19)	NA	.38
Exploratory post hoc analyses^c^					
Children with AOM with a complication, No. (%)^b^^,^^d^	1 (4.2)	2 (5.9)	−1.70 (−15.84 to 15.26)	1.41 (0.20 to 10.51)	.77
Children with tympanostomy tube insertion or adenoidectomy after study entry, No. (%)^e^	4 (1.5)	4 (1.5)	0 (−2.27 to 2.23)	1.00 (−0.16 to 0.16)	.99
No. of days of child’s absence from day care due to symptoms of infection per PYR, median (IQR)^f^	14.90 (4.06 to 28.39)	14.19 (4.06 to 30.42)	−1.90 (−5.33 to 1.54)	NA	.73
Children with respiratory symptoms leading to SARS-CoV-2 testing, No. (%)^b^	188 (45.4)	184 (44.6)	0.90 (−5.91 to 7.62)	1.02 (0.88 to 1.19)	.80
Positive SARS-CoV-2 test result, No. (%)^b^	3 (0.7)	2 (0.5)	0.24 (−1.00 to 1.48)	1.50 (0.30 to 7.49)	.66

Abbreviations: AOM, acute otitis media; PYR, person-years at risk.

^a^ The interpretation of this statistical analysis should be done with caution, as this study was not designed for subgroup analyses.

^b^ Monthly questionnaires used for the secondary outcomes were available for 679 (82.1%) children at 1 month, 653 (79.0%) at 2 months, 617 (74.6%) at 3 months, 600 (72.6%) at 4 months, 576 (69.6%) at 5 months, and 553 (66.9%) at 6 months.

^c^ Data were available from 414 children in the placebo group and 413 in the *Streptococcus salivarius* K12 group.

^d^ Complication was defined as perforation of the tympanic membrane. None of the children had mastoiditis. Includes 24 children in the placebo group and 34 in the *Streptococcus salivarius* K12 group.

^e^ Data were available from 273 children in the placebo group and 270 in the *Streptococcus salivarius* K12 group.

^f^ Data were available from 381 children in the placebo group and 375 in the *Streptococcus salivarius* K12 group.

### Secondary Outcomes

**Figure 2.**
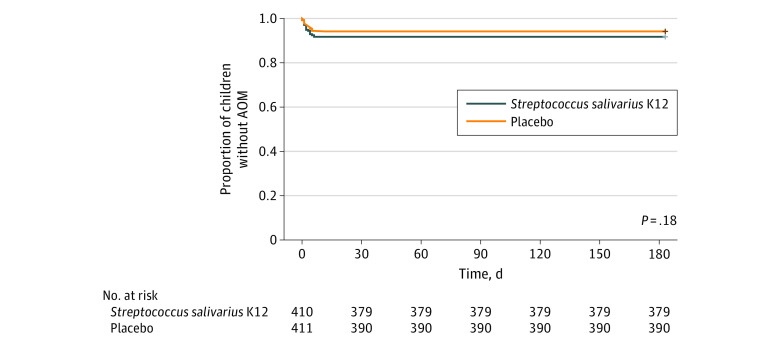
Time to First Episode of Acute Otitis Media (AOM) Requiring Antimicrobial Therapy During the Intervention The *P* value was obtained via a log-rank test for difference between groups.

The mean time to the first episode of AOM was 174 (95% CI, 171-177) days in the *S salivarius* group and 176 (95% CI, 173-179) days in the placebo group (*P* = .18) (Figure 2). Altogether, 4 children (1.0%) in the *S salivarius* group and 2 (0.5%) in the placebo group had recurrent otitis media (proportion difference, −0.50% [95% CI, −1.81% to 0.84%]; *P* = .41) (Table 2). Forty-nine children (11.9%) were receiving any antimicrobial therapy in the *S salivarius* group, as were 37 (8.9%) in the placebo group (proportion difference, −2.93% [95% CI, −7.09% to 1.27%]; *P* = .17). There were no significant differences in the proportion of children with at least 1 physician’s appointment due to an acute illness or in the proportion of children hospitalized for acute respiratory symptoms (Table 2).

The median number of all physician-diagnosed AOM episodes was 0 (IQR, 0-0) per PYR in the *S salivarius* group and 0 (IQR, 0.00-0.00) per PYR in the placebo group (mean difference, −0.14% [95% CI, −0.34% to 0.07%]; *P* = .16) (Table 2). There were no significant differences between the groups in the mean number of days with respiratory or gastroenteritis symptoms or parental absence from work due to the child’s illness.

### Exploratory Post Hoc Analyses

There were no significant differences between groups in the proportion of children with AOM with a complication or in AOM-related surgery (Table 2). The SARS-CoV-2 test result was positive for 2 children (0.5%) in the *S salivarius* group and 3 (0.7%) in the placebo group during the study period. The mean (SD) number of days of a child’s absence from day care was 21.90 (25.66) per PYR in the *S salivarius* group and 19.96 (21.74) per PYR in the placebo group.

## Discussion

In this RCT of 827 children attending day care in the Oulu Region, Finland, daily use of the *S salivarius* K12 oral probiotic product for 6 months did not reduce the occurrence of AOM. Previous studies suggesting the clinical efficacy of *S salivarius* in the prevention of AOM have mainly been conducted with nasal sprays in otitis-prone child populations.^21,22,23^ The incidence of AOM in *S salivarius* K12 was higher than in the placebo group, but the difference was not statistically significant. The incidence of AOM was higher in participants from the *S salivarius* K12 younger than 3 years, but as this study was not designed to have enough power for subgroup analysis, these results need to be interpreted with caution. In the primary prevention of AOM, *S salivarius* K12 oral probiotic products appeared to be ineffective.

Two previous RCTs^11,23^ have investigated the effectiveness of probiotic streptococci in the prevention of AOM. In the first RCT of 130 children aged 6 months to 6 years, an α-streptococcal nasal spray initiated after antimicrobial treatment protected against recurrent AOM compared with the placebo by a reduction of 20%.^11^ Similarly, in another RCT including 100 children aged 1 to 5 years, the use of *S salivarius* 24SMB intranasal spray prevented the occurrence of AOM compared with the placebo.^23^ In contrast to the present study, previous RCTs were conducted among children who had had recurrent otitis media.^11,23^

We chose the oral *S salivarius* product due to its ease of use for different age groups and commercial availability. Although oral *S salivarius* preparations have previously been shown to successfully colonize saliva and may reduce the relative abundance of otopathogens in the nasopharyngeal microbiota,^13^ we cannot rule out the possibility that nasal spray solutions may be superior to oral preparations. An additional difference is that the study by Roos et al^11^ was conducted before the implementation of PCV into national immunization programs. *Streptococcus salivarius* K12 alone may not be sufficient in supporting the nasopharyngeal microbiota, as the other significant otopathogens *Haemophilus influenzae* and *Moraxella catarrhalis* may overcome its inhibitory effects by other direct or indirect methods yet unknown.

Our study was conducted during the COVID-19 pandemic. Finland decided to keep day care centers and primary schools open throughout the study period, which enabled the circulation of rhinoviruses,^24^ the most common viruses associated with AOM.^25^ The incidence rate of AOM before the COVID-19 pandemic was approximately 0.9 to 1.6 per PYR in children aged 0 to 2 years.^4^ The decline in the incidence of respiratory infections and antibiotic prescriptions and, thus, the incidence of AOM was surprisingly steep, approximately 60% to 90% according to current estimates.^19,24,26,27,28,29^ We recalculated the sample size during the study, and although the total number of AOM episodes decreased, the power of this study was sufficient to assess the efficacy of *S salivarius* K12 products in preventing AOM. Despite the very low incidence of AOM, we were able to conduct a relatively precise estimate of the efficacy. With a higher incidence of circulating respiratory viruses and therefore a higher incidence of AOM, it is possible that *S salivarius* K12 might have had a different effect.

### Strengths and Limitations

The strengths of this trial include the randomized study design in a real-life setting to investigate the primary prevention of AOM as diagnosed by primary care physicians. To include AOM episodes not treated with antibiotics, we report all AOM episodes as a secondary outcome, because the national guideline allowed watchful waiting as a treatment strategy during the study.^18^ Baseline information showed balanced characteristics over study groups. The findings can be generalized to the general child population for primary prevention of AOM. An additional strength is that we retrieved data on the antimicrobial purchases from the comprehensive national register and reviewed all the original medical records.

This study also has some limitations. A major limitation is that it was conducted during the COVID-19 pandemic, when there was a very low circulation of several respiratory viruses.^30^ Furthermore, the study was conducted before the emergence of SARS-CoV-2 variants such as the Omicron variant. The SARS-CoV-2 variants may promote the evolution of viral respiratory symptoms to AOM, similarly to the rhinovirus.^31^ Had the study been conducted during circulation of different SARS-CoV-2 variants, with a higher incidence of AOM, it might have yielded different results. In addition, our commercially available chewable tablet for children 3 years or older included *L rhamnosus* GG and *P shermanii* in addition to *S salivarius* K12. These probiotics may have interfered with the probiotic effect of *S salivarius*. However, *L rhamnosus* GG has not been shown to reduce AOM incidence.^32^ Furthermore, variability in AOM diagnostics between primary care physicians likely exists, despite a national guideline for AOM diagnosis and management. The proportion of children younger than 1 year is very low in day care centers in Finland. We opted not to offer this study to these children, as their ability to consume oral powder was uncertain.

## Conclusions

In this RCT, we found that daily use of the *S salivarius* K12 oral probiotic product for 6 months did not reduce the occurrence of AOM in children attending day care. New approaches for the primary prevention of AOM, the most common reason for antibiotic use in young children, are needed.

## Notes

Supplement 1.
Trial Protocol

Supplement 2.
Data Sharing Statement

## References

[1] Vaz LE, Kleinman KP, Raebel MA, . Recent trends in outpatient antibiotic use in children. Pediatrics. 2014;133(3):375-385. doi:10.1542/peds.2013-2903 24488744 PMC3934343

[2] Jokinen J, Palmu AA, Kilpi T. Acute otitis media replacement and recurrence in the Finnish Otitis Media Vaccine trial. Clin Infect Dis. 2012;55(12):1673-1676. doi:10.1093/cid/cis799 22972864

[3] Fireman B, Black SB, Shinefield HR, Lee J, Lewis E, Ray P. Impact of the pneumococcal conjugate vaccine on otitis media. Pediatr Infect Dis J. 2003;22(1):10-16. doi:10.1097/00006454-200301000-00006 12544402

[4] Karppinen S, Toivonen L, Schuez-Havupalo L, . Effectiveness of the ten-valent pneumococcal *Haemophilus influenzae* protein D conjugate vaccine (PHiD-CV10) against all respiratory tract infections in children under two years of age. Vaccine. 2019;37(22):2935-2941. doi:10.1016/j.vaccine.2019.04.026 31027929

[5] Vesikari T, Forsten A, Seppä I, . Effectiveness of the 10-valent pneumococcal nontypeable *Haemophilus influenzae* protein D–conjugated vaccine (PHiD-CV) against carriage and acute otitis media—a double-blind randomized clinical trial in Finland. J Pediatric Infect Dis Soc. 2016;5(3):237-248. doi:10.1093/jpids/piw010 27125273 PMC5125453

[6] Norhayati MN, Ho JJ, Azman MY. Influenza vaccines for preventing acute otitis media in infants and children. Cochrane Database Syst Rev. 2017;10(10):CD010089. doi:10.1002/14651858.CD010089.pub3 29039160 PMC6485791

[7] Heikkinen T, Ruuskanen O, Waris M, Ziegler T, Arola M, Halonen P. Influenza vaccination in the prevention of acute otitis media in children. AJDC. 1991;145(4):445-448. doi:10.1001/archpedi.1991.02160040103017 1849344

[8] Tapiovaara L, Lehtoranta L, Swanljung E, . *Lactobacillus rhamnosus* GG in the middle ear after randomized, double-blind, placebo-controlled oral administration. Int J Pediatr Otorhinolaryngol. 2014;78(10):1637-1641. doi:10.1016/j.ijporl.2014.07.011 25085073

[9] Lehtoranta L, Pitkäranta A, Korpela R. Probiotics in respiratory virus infections. Eur J Clin Microbiol Infect Dis. 2014;33(8):1289-1302. doi:10.1007/s10096-014-2086-y 24638909 PMC7088122

[10] Chonmaitree T, Jennings K, Golovko G, . Nasopharyngeal microbiota in infants and changes during viral upper respiratory tract infection and acute otitis media. PLoS One. 2017;12(7):e0180630. doi:10.1371/journal.pone.0180630 28708872 PMC5510840

[11] Roos K, Håkansson EG, Holm S. Effect of recolonisation with “interfering” alpha streptococci on recurrences of acute and secretory otitis media in children: randomised placebo controlled trial. BMJ. 2001;322(7280):210-212. doi:10.1136/bmj.322.7280.210 11159619 PMC26587

[12] Power DA, Burton JP, Chilcott CN, Dawes PJ, Tagg JR. Preliminary investigations of the colonisation of upper respiratory tract tissues of infants using a paediatric formulation of the oral probiotic *Streptococcus salivarius* K12. Eur J Clin Microbiol Infect Dis. 2008;27(12):1261-1263. doi:10.1007/s10096-008-0569-4 18560907

[13] Sarlin S, Tejesvi MV, Turunen J, . Impact of *Streptococcus salivarius* K12 on nasopharyngeal and saliva microbiome: a randomized controlled trial. Pediatr Infect Dis J. 2021;40(5):394-402. doi:10.1097/INF.0000000000003016 33298762 PMC8043514

[14] Zupancic K, Kriksic V, Kovacevic I, Kovacevic D. Influence of oral probiotic *Streptococcus salivarius* K12 on ear and oral cavity health in humans: a systematic review. Probiotics Antimicrob Proteins. 2017;9(2):102-110. doi:10.1007/s12602-017-9261-2 28236205

[15] Lévesque C, Lamothe J, Frenette M. Coaggregation of *Streptococcus salivarius* with periodontopathogens: evidence for involvement of fimbriae in the interaction with *Prevotella intermedia*. Oral Microbiol Immunol. 2003;18(5):333-337. doi:10.1034/j.1399-302X.2003.00085.x 12930529

[16] Kumpu M, Kekkonen RA, Kautiainen H, . Milk containing probiotic *Lactobacillus rhamnosus* GG and respiratory illness in children: a randomized, double-blind, placebo-controlled trial. Eur J Clin Nutr. 2012;66(9):1020-1023. doi:10.1038/ejcn.2012.62 22692023

[17] Kanta services. Accessed February 28, 2023. https://www.kanta.fi/

[18] Finnish Medical Society Duodecim. Current care guidelines: acute otitis media. September 6, 2017. Accessed September 17, 2022. https://www.kaypahoito.fi/

[19] Kuitunen I, Artama M, Mäkelä L, Backman K, Heiskanen-Kosma T, Renko M. Effect of social distancing due to the COVID-19 pandemic on the incidence of viral respiratory tract infections in children in Finland during early 2020. Pediatr Infect Dis J. 2020;39(12):e423-e427. doi:10.1097/INF.0000000000002845 32773660

[20] Armitage P, Berry G, Matthews JNS. Statistical Methods in Medical Research. 4th ed. Wiley-Blackwell; 2020.

[21] Di Pierro F, Di Pasquale D, Di Cicco M. Oral use of *Streptococcus salivarius* K12 in children with secretory otitis media: preliminary results of a pilot, uncontrolled study. Int J Gen Med. 2015;8:303-308. doi:10.2147/IJGM.S92488 26396541 PMC4576902

[22] Di Pierro F, Colombo M, Giuliani MG, . Effect of administration of *Streptococcus salivarius* K12 on the occurrence of streptococcal pharyngo-tonsillitis, scarlet fever and acute otitis media in 3 years old children. Eur Rev Med Pharmacol Sci. 2016;20(21):4601-4606. 27874935

[23] Marchisio P, Santagati M, Scillato M, . *Streptococcus salivarius* 24SMB administered by nasal spray for the prevention of acute otitis media in otitis-prone children. Eur J Clin Microbiol Infect Dis. 2015;34(12):2377-2383. doi:10.1007/s10096-015-2491-x 26385346

[24] Kuitunen I, Renko M. Antibiotic prescriptions during the first 2 years of the COVID-19 pandemic in Finnish children. Acta Paediatr. 2023;112(1):143-145. doi:10.1111/apa.16515 35982603 PMC9538447

[25] Arola M, Ziegler T, Ruuskanen O, Mertsola J, Näntö-Salonen K, Halonen P. Rhinovirus in acute otitis media. J Pediatr. 1988;113(4):693-695. doi:10.1016/S0022-3476(88)80380-9 2845041 PMC7173229

[26] Kadambari S, Goldacre R, Morris E, Goldacre MJ, Pollard AJ. Indirect effects of the COVID-19 pandemic on childhood infection in England: population based observational study. BMJ. 2022;376:e067519. doi:10.1136/bmj-2021-067519 35022215 PMC8753487

[27] Marom T, Schwarz Y, Gluck O, Ginzburg G, Tamir SO. Trends in pediatric acute otitis media burden during the first COVID-19 year. Otol Neurotol. 2022;43(7):e760-e766. doi:10.1097/MAO.0000000000003581 35878638

[28] Marom T, Pitaro J, Shah UK, . Otitis media practice during the COVID-19 pandemic. Front Cell Infect Microbiol. 2022;11:749911. doi:10.3389/fcimb.2021.749911 35071032 PMC8777025

[29] Hullegie S, Schilder AGM, Marchisio P, . A strong decline in the incidence of childhood otitis media during the COVID-19 pandemic in the Netherlands. Front Cell Infect Microbiol. 2021;11:768377. doi:10.3389/fcimb.2021.768377 34790591 PMC8591181

[30] Kuitunen I, Artama M, Haapanen M, Renko M. Rhinovirus spread in children during the COVID-19 pandemic despite social restrictions—a nationwide register study in Finland. J Med Virol. 2021;93(10):6063-6067. doi:10.1002/jmv.27180 34228369 PMC8426983

[31] Franch-Llasat D, Bellaubí-Pallarés N, Pérez-Moreno MO, Chamarro-Martí E, García-Rodríguez E, Roche-Campo F. Pneumococcal meningitis secondary to otitis media in two patients with COVID-19 Omicron variant. Int J Emerg Med. 2022;15(1):50. doi:10.1186/s12245-022-00448-y 36104658 PMC9472739

[32] Hatakka K, Blomgren K, Pohjavuori S, . Treatment of acute otitis media with probiotics in otitis-prone children—a double-blind, placebo-controlled randomised study. Clin Nutr. 2007;26(3):314-321. doi:10.1016/j.clnu.2007.01.003 17353072

