# Errors in Table

**DOI:** 10.1001/jamanetworkopen.2023.52223

**Published:** 2023-12-27

In the Original Investigation titled “*Streptococcus salivarius* Probiotics to Prevent Acute Otitis Media in Children: A Randomized Clinical Trial,”^1^ published November 2, 2023, the following errors occurred in Table 2: for the duration of hospitalization outcome, the median (IQR) of the proportion or mean difference and the *P* value were missing; the median for the runny nose questionnaire response in the intervention group was incorrect; and the values for the No. of days of parental absence were incorrectly described as the mean (SD) instead of the median (IQR). This article has been corrected.
