# Supplementary material for: Streptococcus salivarius Probiotics to Prevent Acute Otitis Media in Children: A Randomized Clinical Trial
Source: JAMA Netw Open. 2023 Nov 2;6(11):e2340608. doi: 10.1001/jamanetworkopen.2023.40608 (PMC10623191; doi:10.1001/jamanetworkopen.2023.40608)
Supplement: Supplement 1. — Trial Protocol [file jamanetwopen-e2340608-s001.pdf]

Effect of *Streptococcus salivarius* K12 Oral Probiotic Products on Prevention of Acute Otitis Media in Children: A Randomized Clinical Trial

ORIGINAL RESEARCH PROTOCOL AND STATISTICAL ANALYSIS PLAN

Suvi Sarlin, M.D.[1,2], Ulla Koskela, M.D., Ph.D.[1,2,3], Minna Honkila, M.D., Ph.D.[1,2], Paula Tähtinen, M.D., Ph.D.[4] Tytti Pokka, M.Sc.[1,2,5], Marjo Renko, M.D., Ph.D.[6], Terhi Tapiainen, M.D., Ph.D.[1,2,7]

1. Department of Pediatrics and Adolescent Medicine, Oulu University Hospital, Oulu, Finland
2. Research Unit of Clinical Medicine and Medical Research Center Oulu, University of Oulu, Oulu, Finland
3. Department of Anesthesiology, Oulu University Hospital, Finland
4. Department of Pediatrics, University of Turku, Turku, Finland
5. Research Service Unit, Oulu University Hospital, Finland
6. University of Eastern Finland and Kuopio University Hospital, Kuopio, Finland
7. Biocenter Oulu, University of Oulu, Oulu, Finland

[#]**Address correspondence to:** Terhi Tapiainen, Department of Pediatrics and Adolescent Medicine, Oulu University Hospital, P.O. Box 23, FIN 90029 Oulu, Finland, [suvi.sarlin@oulu.fi], telephone: +358-8-3158426, fax: +358-8-3155559

Alternative correspondent: Suvi Sarlin, Department of Pediatrics and Adolescent Medicine, Oulu University Hospital, P.O. Box 23, FIN 90029 Oulu, Finland, [terhi.tapiainen@oulu.fi], telephone: +358-8-3155185, fax: +358-8-31555

ROLES

Concept and design: Koskela, Sarlin, Tähtinen, Renko, Tapiainen.

Acquisition, analysis, or interpretation of data: All authors.

Drafting of the manuscript: Koskela, Sarlin, Honkila, Tapiainen.

Critical revision of the manuscript for important intellectual content: All authors.

Statistical analysis: Sarlin, Honkila, Pokka, Tapiainen.

Funding obtained: Sarlin, Tapiainen.

Supervision: Honkila, Tähtinen, Renko, Tapiainen.

ABBREVIATIONS

AOM	acute otitis media
CFUs	colony-forming units
CI	confidence interval
MEE	middle ear effusion
PCV7	7-valent pneumococcal conjugate vaccine with 7 pneumococcal serotypes included
PCV10	10-valent pneumococcal *Haemophilus influenzae* protein D conjugate vaccine with 10 pneumococcal serotypes included
RR	risk ratio

1. BACKGROUND, RATIONALE, AND HYPOTHESIS

Background

Acute otitis media (AOM) is the most common reason for antibiotic use for young children.[1] The common bacterial otopathogens *Streptococcus pneumoniae*, *Haemophilus influenzae*, and *Moraxella catarrhalis* are found in the nasopharynx from early infancy.[2,3] A viral respiratory tract infection enhances changes in the nasopharyngeal environment, resulting in the increased adhesion and growth of otopathogens and their rise via Eustachian tube from the oral cavity and nasopharynx to the middle ear.[4-6] Current options for primary prevention of AOM are limited. The pneumococcal conjugate vaccine (PCV) only modestly decreases the incidence of AOM, with estimates ranging from 8% to 10% for PCV7[7,8] and from no effect to 23% for PCV10.[9,10] Influenza vaccination reduces the occurrence of AOM during the influenza epidemic season.[11,12] Xylitol products are effective in preventing AOM only if administered regularly after every meal.[13-16] Thus, novel options for the primary prevention of AOM are needed.

Rationale

Probiotics containing *Lactobacillus rhamnosus* GG may be effective in preventing symptomatic viral respiratory infections.[17,18] Lactobacilli do not, however, belong to the nasopharyngeal core microbiome.[19] Accordingly, probiotic lactobacilli have proven ineffective in AOM prevention,[20] even when *L. rhamnosus* GG nasopharyngeal colonization has been achieved.[21] In contrast to lactobacilli, alpha-hemolytic streptococci belong to the normal core microbiome of the nasopharynx.[22-24] In an earlier study in Sweden, an in-house alpha-streptococcal mixture spray after the antimicrobial treatment of AOM successfully reduced the recurrence of AOM.[25] The in-house bacterial sprays have not been evaluated for their safety and may vary in their efficacy. Thus, they are not suitable for large interventions.

Commercially available probiotic products containing *Streptococcus salivarius* strains K12, M18, or 24smb have recently been developed for oral health care.[26-29] *S. salivarius* K12 was originally isolated from the oral cavity of a healthy schoolboy in New Zealand.[29] *S. salivarius* K12 uses bacteria–bacteria and host–bacteria contacts and indirect methods to defend its habitat.[30] *S. salivarius* K12 produces bacteriocin-like inhibitory substances, including salivaricins. *S. salivarius* K12 oral probiotic products have been shown to successfully colonize the human oral cavity and pharynx[31,32] and to produce the lantibiotic salivaricin.[33] Previous clinical studies using *S. salivarius*

K12 products mainly focused on streptococcal pharyngotonsillitis, with some favorable results.[34-37] Earlier, we showed that oral *S. salivarius* K12 products (oral soluble powder or chewable tablets) reduced the relative abundance of otopathogens in the nasopharyngeal microbiome in a randomized trial (Sarlin S. submitted manuscript, EudraCT 2017-000820-83). The diversity of the nasopharyngeal microbiome remained unchanged. Families and children found both products feasible to use.

In this randomized, placebo-controlled trial, we will investigate the clinical efficacy of *S. salivarius* K12 probiotic products in preventing AOM in children.

2. METHODS

Study design and study population

This study is designed as a pragmatic, placebo-controlled, double-blind, randomized (parallel allocation ratio 1:1) clinical trial in young children attending day care centers in the City of Oulu, Finland. The children will be randomly allocated to receive either an oral *S. salivarius* K12 product or placebo. Young children (< 3 years of age) will use oral powder, and older children (> 3 years of age) will use chewing tablets. The products have been used in a randomized clinical trial showing the microbiological effect in the nasopharyngeal microbiome (Sarlin S. submitted manuscript, EudraCT 2017-000820-83).

Recruitment

Participation will be offered to all children attending day care centers in Oulu in August–November 2020. Intervention and clinical follow-up will last 6 months from study entry. Day-care children have an increased risk of AOM.[13] Our aim is to recruit children when they have started day care after the summer holiday and before they acquire their first viral respiratory infection.

Only children whose families have given their written informed consent will be enrolled. To inform parents, study physicians will visit the evening sessions of day-care centers and give presentations about the rationale of the study, at which parents will be allowed to ask questions. Study physicians will visit day-care centers and examine the ear status of the children before recruiting.

Addition 8.9.2020. Due to the COVID-19 pandemic and restrictions in the day-care centers, the ear status of children will not be examined. Local child health clinics will share study information

pamphlets for the parents. AOM infections during the last 6 months will be inquired about on the electronic background data sheet. Informed consent forms will be gathered from day-care centers or via mail. Study physicians will deliver the study products either to day-care centers or personally. Parents can contact the study physicians any time via email, call, or text messages or in weekly open Zoom meetings.

Addition 30.10.2020. Due to small attendance, we expanded the recruitment area to municipal or local private day-care centers Muhos, Liminka, and Muhos and Kempele in the Oulu region, Finland.

Inclusion criteria

- Age 12 months–7 years
- Day care attendance or a younger sibling of study participant who is not yet attending day care
- Written informed consent from a parent
- Able to use oral study products

Exclusion criteria

- Middle ear effusion (MEE) at study entry (assessed by tympanometry and/or otoscopy by study physician)
- Ongoing continuous antimicrobial prophylaxis
- Immunosuppression or primary immunodeficiency, including Down's syndrome
- The use of other probiotic products is discouraged but is not used as an exclusion criterion

Addition 8.9.2020. Due to the COVID-19 pandemic and restrictions in day-care centers, the ear status of children will not be examined. Thus, MEE has been removed from the exclusion criteria.

Interventions

Children will receive an oral probiotic product **every evening for 6 months**. One daily dose is one sachet of **oral soluble powder** for young children (< 3 y) and **one chewable tablet** for older children (≥ 3 y). *S. salivarius* chewable tablets are available in Finland as a commercial over-the-counter probiotic product (ToothGuide®, GutGuide Ltd., Finland). A daily dose contains **1 × 10^9 colony-forming units (CFUs) of *S. salivarius* K12**, a quantity that has previously been successful in the colonization of the nasopharynx in adults.[36]

Products for children < 3 years of age

- One sachet of soluble oral powder in the treatment arm contains 1 x 10^9 CFUs of *S. salivarius* K12 per sachet with 1010 mg of maltodextrin (bulking agent), fructo-oligosaccharide (bulking agent), and strawberry flavors (10 mg).
- One sachet of soluble oral powder in the placebo arm looks and tastes similar to the treatment product. The placebo contains 1010 mg of maltodextrin (bulking agent), FOS (bulking agent), and strawberry flavors (10 mg) without *S. salivarius* K12.

Addition 30.10.20

- Sachets are also suitable for children > 3 years of age.

Products for children ≥ 3 years of age

- One oral chewable table in the treatment arm contains 1 x 10^9 CFUs of *S. salivarius* K12 with isomaltitol (789 mg), xylitol (72.2 mg), peppermint flavor (10 mg), silicon dioxide (6.9 mg), D3 vitamin (10 µg), *Lactobacillus rhamnosus* GG (1 x 10^8 CFUs), and *Propionibacterium shermanii* (1 x 10^8 CFUs).
- The chewable tablets in the placebo arm look and taste similar. The placebo chewable tablet contains all the same ingredients, including D3 vitamin, but without *S. salivarius* K12, *Lactobacillus rhamnosus* GG, and *Propionibacterium shermanii* (1 x 10^8 CFUs).

Randomization

The randomization lists and sheets will be created by a biostatistician—who will not participate in the recruitment or clinical follow-up—with **computerized block randomization using permuted blocks of variable size.** Randomization with a 1:1 allocation ratio will be stratified according to

age (children < 3 years and ≥ 3 years). The individual randomization sheets will be inserted into opaque envelopes with ascending numbers on the cover. The study physician will open each sealed randomization envelope after the parent has signed the written informed consent and the ears have been examined to rule out MEE at study entry.

Primary outcome

- The proportion of children with at least one AOM episode requiring antimicrobial treatment in 0–6 months

Secondary outcomes

- The proportion of children with a recurring AOM episode requiring antimicrobial treatment in 0–6 months (i.e., at least 3 AOM episodes in 0–6 months)
- Time to the first AOM episode requiring antimicrobial treatment during the intervention until 6 months
- The incidence density of all AOM episodes diagnosed by physician (episodes of AOM per PYR, person years at risk) in 0–6 months
- The proportion of children with any antimicrobial treatment in 0–6 months
- The proportion of children with any physician appointments due to acute illness in 0–6 months
- The number of new acute respiratory infections in 0-6 months
- Number of days of parental absenteeism from work due to a child's illness in 0–6 months

Addition 28.5.2020

- The proportion of children with hospitalization due to acute respiratory illness in 0–6 months and duration of hospitalization

Addition 30.10.2020 Due to the COVID-19 pandemic, large-scale respiratory sampling from all children with any respiratory symptoms has been recommended in Finland from August 2020 onward. For these reasons, we added the following outcome variables to the research protocol:

- Proportion of children with a respiratory viral sample (i.e., respiratory symptoms in 0–6 months)
- Proportion of children with COVID-19 infection or positive SARS-CoV-2 sample in 0–6

months

- Proportion of children with throat swab
- Proportion of children with *Streptococcus pyogenes* infection or positive StrepA culture in 0–6 months
- Proportion of children with other bacterial findings or viral findings in 0–6 months

Addition 17.12.21 We added following outcomes to the research protocol:

- Proportion of children with at least one respiratory tract infection episode in 0–6 months
- Proportion of children who have undergone COVID-19 testing in 0–6 months
- The number of any AOM episodes per child (including parent-reported AOM episodes without antibiotic prescriptions) in 0–6 months
- Proportion of children with ≥ 1 acute respiratory tract infection episode in 0–6 months
- Number of acute respiratory tract symptom days per child in 0–6 months
- The proportion of children with hospitalization due to acute respiratory illness in 0–6 months and duration of hospitalization for any acute reason
- Proportion of children with ≥ 1 parent who has reported wheezing episodes in 0–6 months
- Incidence of acute AOM episodes measured as verified and purchased antibiotic prescriptions in 0–6 months
- Incidence of acute AOM episodes measured as parent reported episodes or AOM episodes that passed with watchful waiting and verified antibiotic prescriptions in 0–6 months

We report the numbers of the following samples without comparing them between groups, thus removing them from secondary outcomes:

- Proportion of children with a respiratory viral sample (i.e. respiratory symptoms in 0–6 months)
- Proportion of children with a throat swab
- Proportion of children with *Streptococcus pyogenes* infection or positive StrepA culture in 0–6 months
- Proportion of children with other bacterial findings or viral findings in 0–6 months

Sample size calculation

We will compare the clinical efficacy of *S. salivarius* K12-containing products in preventing AOM in day-care children with that of placebo products. The primary outcome is the proportion of children with at least AOM episode during the study.

The **baseline** for the proportion of day-care children with at least one AOM episode is predicted to be **30%** during the 6-month trial (Sep–Feb). The estimate is based on the earlier publications before and after the pneumococcal conjugate vaccine was implemented in the national vaccination program in Finland in 2010. In 2006, altogether, 20% of Finnish day-care children (1–6 y of age) in the city of Oulu experienced at least one AOM episode in 3 months.[16] In another cohort of young Finnish children, approximately 800 AOM episodes occurred in 1000 children in 6 months during the epidemic season (September–February).[38] In the pneumococcal conjugate vaccine era, Finnish children with the pneumococcal vaccine had 1.0 AOM episode per one year, as compared with 1.3 episodes per year in those unvaccinated.[9]

We regard the effect to be clinically significant if the occurrence of AOM decreases by 30% (i.e., the proportion of children **with at least one AOM episode decreases from 30% to 21% in 6 months).** With a statistical power of 80% and alpha error of 5%, **we will recruit 389 children per group (salivarius vs. placebo).** To compensate for a possible 5% rate of dropouts and lacking clinical information, we will recruit at least 410 children per group. For children younger than 3 years of age, we will use oral powder sachets **(*38 000 treatment and 38 000 placebo sachets available***; products available for 211 children per group), and for older children, chewable tablets **(*1800 x 30 chewable tablets for treatment and 1800 x 30 for the placebo group available***; products available for 300 children per group). Altogether, **we will recruit at least 820 children** but no more than 1000 children.

Addition 30.10.2020: *Due to the COVID-19 pandemic*, the occurrence of AOM is likely to decrease due to large-scale hygiene interventions in society. We assume that the proportion of children with at least one AOM will decrease from 30% to 12% (60% relative decrease). With this new baseline occurrence, we regard a 50% relative decrease as clinically significant **(12% to 6%),** with 80% power and 5% alpha error. The required novel sample size is 356 per group (uncorrected chi-square) and 389 per group (corrected chi-square), in total 778 children. As the primary outcome

will be available for all children from the comprehensive national registry, we assume a small dropout rate of 3%. Thus, we will recruit **at least 801** children.

Definitions, clinical follow-up, and data collection

AOM diagnosis is defined according to the national Finnish Current Clinical Guideline for AOM as an acute, short-term, and clinically diagnosed episode of otitis media, with signs of tympanic membrane inflammation and the presence of MEE and at least one acute clinical symptom, such as rhinitis, cough, fever, throat ache, ear ache, impaired hearing, or increased crying.[39]

Background characteristics will be solicited through an electronic questionnaire at study entry. Background characteristics include the known risk factors for AOM: the duration of day care, the number of siblings, parental smoking, previous history of AOM and related operations, presence of ventilation tubes, the duration of breastfeeding, the previous or current use of a pacifier, previous vaccinations, current medications, previous and current use of probiotic products, and any antimicrobial treatment during the preceding 6 months before study entry.

The primary outcome will be met (i.e., AOM requiring antimicrobial treatment will be recorded) if a parent has purchased antimicrobial prescription on the comprehensive national prescription registry (Kanta.fi) by any primary care physician or hospital unit and the review of original medical records confirms that the indication for the antimicrobial treatment was AOM, defined according to the national guideline.

For secondary outcomes, recurring AOM episodes requiring antimicrobial treatment and the time to the first AOM episode requiring antimicrobial treatment will be defined and recorded in a similar manner as the primary outcome. Any AOM diagnosis, regardless of antimicrobial treatment, made by any physician, will be solicited through **monthly web-based questionnaires** administered to families and by review of medical records. Any antimicrobial treatment is defined as any course of antimicrobial treatment regardless of the prescription indication. The number of days with any antimicrobial treatment will be recorded from the comprehensive national prescription registry (Kanta.fi). Any physician appointments and novel and parental absenteeism from work due to their child’s illness will be solicited through monthly web-based questionnaires.

The compliance and the use of other probiotic products during the study will be collected through monthly web-based questionnaires.

Time schedule

- March–April 2020: ethical committee approval and Fimea (Finnish Medicines Agency) approval with EudraCT number and the research approval by the City of Oulu
- March–April 2020: web-based questionnaires and electronic symptom sheet diaries
- May 2020: trial registration on ClinicalTrials.gov
- August–October 2020: recruiting study physicians Dr. Ulla Koskela and Dr. Suvi Sarlin to visit 50 day care centers
- Intervention and follow-up 6 months from Aug–Oct 2020 to Feb–May 2021.
    - Monthly questionnaires and retrieval of electronic symptom sheet diary data by study nurse Leena Okkonen
    - Review of the medical records of all children with antimicrobial use during the study by Dr. Ulla Koskela and Dr. Suvi Sarlin

Addition 30.9.2020. An electronic AOM diagnostic helper with 8 ear status pictures, including purulent AOM, will be sent to parents. If AOM is diagnosed, the parents will present the pictures to the attending doctor. The doctor will add the number of pictures best representing the ear status to the patient records.

- The electronic diary will include 8 different pictures of healthy tympanum, bulging and infected tympanum, and tympanum with chronic otitis media effusion. If AOM is diagnosed, parents will present the pictures to the attending doctor, who will add the most presentative pictures to the patient records.

Addition 30.10.2020. Due to small attendance, we expanded the recruitment area to municipal or local private day-care centers Muhos, Liminka, and Muhos and Kempele in the Oulu region, Finland.

Statistical analysis plan

All analyses will be conducted on the intention-to-treat population (i.e., in all randomized patients regardless of their compliance with the study product). Only outcomes specified in the protocol before data analysis will be compared. We will compare the proportions of children who meet the primary outcome or secondary outcomes between groups by the standard normal deviate test and will present 95% confidence intervals (CIs) of the differences. In addition to absolute risk reduction, risk ratios with 95% CIs will be calculated. Time-to-event analysis will be performed

using the Kaplan-Meier estimator and tested by a log-rank test. The number of days with parental absenteeism will be compared using a t-test or Mann–Whitney U-test as applicable, and the 95% CIs of the differences will be reported. If there is a statistically significant difference in the primary outcome between the groups, subgroup analyses in children younger than 3 years and in children older than 3 years will be performed.

3. IMPACT OF THE STUDY

Preventing AOM would be the most convenient and efficient way to reduce problems related to AOM morbidity. The discomfort due to acute symptoms, parental absenteeism from work, and other direct and indirect costs of otitis media are important reasons to prevent AOM in children. AOM has a significant impact on public health as otitis media is the most common reason for antibiotic treatment in young children.[40] Nasopharyngeal pneumococcal carriage is common in children, and children are exposed to antibiotics repeatedly due to frequent otitis media episodes. Antibiotic consumption for common respiratory infections is related to the emerging antimicrobial resistance.[41,42] Furthermore, antimicrobial treatment in children has been reported to be associated with subsequent overweight, juvenile rheumatoid arthritis, inflammatory bowel disease, and asthma.[43-46] This has been suggested to be related to the reported changes in the gut microbiome after oral antimicrobial courses in children.[47] Recurrent otitis media is still the most common reason for surgery in children. In total, 700 000 annual myringotomies with insertions of ventilator tubes are performed in children in the United States.[48] Long-term sequelae of AOM have been suggested because MEE during AOM episode impairs hearing in children, corresponding to hearing levels ranging from 20 to 50 dB.[49] Even though antimicrobial treatment markedly reduces the duration of MEE and hearing impairment due to AOM,[50] children with a prolonged presence of MEE may suffer from reduced receptive and expressive language skills.[51]

4. FUNDING

This is an independent, investigator-driven clinical randomized trial. The study products are purchased via GutGuide Ltd., Finland. The accuracy of the product information in the study protocol was reviewed by the manufacturers before the study. The study is funded by Academy of Finland (to Terhi Tapiainen), Pediatric Research Foundation Finland (to Terhi Tapiainen), and University of

Oulu Graduate School (to Suvi Sarlin). Manufacturers of the products or funders of the study do not interfere with the study protocol, analyses, or writing of the manuscript.

5. ETHICAL CONSIDERATIONS

The study will be conducted according to good clinical practice (GCP) guidelines. All key personnel have completed their GCP training. Only children whose parents give their written informed consent will be recruited. The Ethical Committee of the Oulu University Hospital District will review the study protocol and informed consent forms before the study.

6. TRIAL REGISTRATION

The trial will be registered at ClinicalTrials.gov and ClinicalTrialsRegister.eu before the study.

7. FINNISH MEDICAL AGENCY

The trial will be reported to FIMEA, the representative of the European Medicines Agency, before the study.

References

1. **Vaz LE, Kleinman KP, Raebel MA, et al.** Recent trends in outpatient antibiotic use in children. *Pediatrics*. 2014;**133:**375-385. doi: 10.1542/peds.2013-2903.

2. **Faden H, Duffy L, Wasielewski R, Wolf J, Krystofik D, Tung Y.** Relationship between nasopharyngeal colonization and the development of otitis media in children. Tonawanda/Williamsville Pediatrics. *J. Infect. Dis.* 1997;**175:**1440-1445.

3. **Kilpi T, Herva E, Kaijalainen T, Syrjanen R, Takala AK.** Bacteriology of acute otitis media in a cohort of Finnish children followed for the first two years of life. *Pediatr. Infect. Dis. J.* 2001;**20:**654-662.

4. **Faden H, Stanievich J, Brodsky L, Bernstein J, Ogra PL.** Changes in nasopharyngeal flora during otitis media of childhood. *Pediatr. Infect. Dis. J.* 1990;**9:**623-626.

5. **Revai K, Mamidi D, Chonmaitree T.** Association of nasopharyngeal bacterial colonization during upper respiratory tract infection and the development of acute otitis media. *Clin. Infect. Dis.* 2008;**46:**e34-7. doi: 10.1086/525856.

6. **Chonmaitree T, Jennings K, Golovko G, et al.** Nasopharyngeal microbiota in infants and changes during viral upper respiratory tract infection and acute otitis media. *PLoS One*. 2017;**12:**e0180630. doi: 10.1371/journal.pone.0180630.

7. **Fireman B, Black SB, Shinefield HR, Lee J, Lewis E, Ray P.** Impact of the pneumococcal conjugate vaccine on otitis media. P*ediatr. Infect. Dis. J.* 2003;**22:**10-16. doi: 10.1097/00006454-200301000-00006.

8. **Jokinen J, Palmu AA, Kilpi T.** Acute otitis media replacement and recurrence in the Finnish otitis media vaccine trial. *Clin. Infect. Dis.* 2012;**55:**1673-1676. doi: 10.1093/cid/cis799.

9. **Karppinen S, Toivonen L, Schuez-Havupalo L, et al.** Effectiveness of the ten-valent pneumococcal Haemophilus influenzae protein D conjugate vaccine (PHiD-CV10) against all respiratory tract infections in children under two years of age. *Vaccine*. 2019;**37:**2935-2941. doi: S0264-410X(19)30477-3.

10. **Vesikari T, Forsten A, Seppa I, et al.** Effectiveness of the 10-valent Pneumococcal Nontypeable Haemophilus influenzae protein D-conjugated vaccine (PHiD-CV) against carriage and acute otitis media-A double-blind randomized clinical trial in Finland. *J. Pediatric Infect. Dis. Soc.* 2016;**5:**237-248. doi: 10.1093/jpids/piw010.

11. **Heikkinen T, Block SL, Toback SL, Wu X, Ambrose CS.** Effectiveness of intranasal live attenuated influenza vaccine against all-cause acute otitis media in children. *Pediatr. Infect. Dis. J.* 2013;**32:**669-674. doi: 10.1097/INF.0b013e3182840fe7.

12. **Norhayati MN, Ho JJ, Azman MY.** Influenza vaccines for preventing acute otitis media in infants and children. *Cochrane Database Syst. Rev.* 2017;**10:**CD010089. doi: 10.1002/14651858.CD010089.pub3.

13. **Uhari M, Kontiokari T, Koskela M, Niemela M.** Xylitol chewing gum in prevention of acute otitis media: double blind randomised trial. *BMJ*. 1996;**313:**1180-1184.

14. **Uhari M, Kontiokari T, Niemela M.** A novel use of xylitol sugar in preventing acute otitis media. *Pediatrics*. 1998;**102:**879-884.

15. **Tapiainen T, Luotonen L, Kontiokari T, Renko M, Uhari M.** Xylitol administered only during respiratory infections failed to prevent acute otitis media. *Pediatrics*. 2002;**109:**E19.

16. **Hautalahti O, Renko M, Tapiainen T, Kontiokari T, Pokka T, Uhari M.** Failure of xylitol given three times a day for preventing acute otitis media. *Pediatr. Infect. Dis. J.* 2007;**26:**423-427. doi: 10.1097/01.inf.0000259956.21859.dd.

17. **Hatakka K, Savilahti E, Ponka A, et al.** Effect of long term consumption of probiotic milk on infections in children attending day care centres: double blind, randomised trial. *BMJ*. 2001;**322:**1327.

18. **Lehtoranta L, Pitkaranta A, Korpela R.** Probiotics in respiratory virus infections. *Eur. J. Clin. Microbiol. Infect. Dis.* 2014;**33:**1289-1302. doi: 10.1007/s10096-014-2086-y.

19. **Igartua C, Davenport ER, Gilad Y, Nicolae DL, Pinto J, Ober C.** Host genetic variation in mucosal immunity pathways influences the upper airway microbiome. *Microbiome*. 2017;**5:**16-016-0227-5. doi: 10.1186/s40168-016-0227-5.

20. **Kumpu M, Kekkonen RA, Kautiainen H, et al.** Milk containing probiotic Lactobacillus rhamnosus GG and respiratory illness in children: a randomized, double-blind, placebo-controlled trial. *Eur. J. Clin. Nutr.* 2012;**66:**1020-1023. doi: 10.1038/ejcn.2012.62.

21. **Tapiovaara L, Lehtoranta L, Swanljung E, et al.** Lactobacillus rhamnosus GG in the middle ear after randomized, double-blind, placebo-controlled oral administration. *Int. J. Pediatr. Otorhinolaryngol.* 2014;**78:**1637-1641. doi: 10.1016/j.ijporl.2014.07.011.

22. **Bill NJ, Washington JA.** Bacterial interference by Streptococcus salivarius. *Am. J. Clin. Pathol.* 1975;**64:**116-120.

23. **Brook I.** The role of bacterial interference in otitis, sinusitis and tonsillitis. *Otolaryngol. Head. Neck. Surg.* 2005;**133:**139-146. doi: S0194599805002573.

24. **Tano K, Hellstrom S.** Bacterial adherence to pharyngeal cells: in vitro studies with alpha-haemolytic streptococci and Haemophilus influenzae. *Acta Otolaryngol.* 2002;**122:**745-751.

25. **Roos K, Hakansson EG, Holm S.** Effect of recolonisation with “interfering” alpha streptococci on recurrences of acute and secretory otitis media in children: randomised placebo controlled trial. *BMJ.* 2001;**322:**210-212.

26. **Burton J, Cowley S, Simon R, McKinney J, Wescombe P, Tagg J.** Evaluation of safety and human tolerance of the oral probiotic *Streptococcus salivarius: a* randomized, placebo-controlled, double-blind study. *Food Chem. Toxicol.* 2011;**49:**2356-2346.

27. **Burton J, Drummond B, Chilcott C, et al.** Influence of the probiotic *Streptococcus salivarius* strain M18 on indices of dental health in children: a randomized double-blind, placebo-controlled trial. *J. Med. Microbiol.* 2013;**62:**875-884.

28. **Marchisio P, Santagati M, Scillato M, Baggi E, Fattizzo M, Rosazza C.** Streptococcus salivarius 24SMB administered by nasal spray for the prevention of acute otitis media in otitis-prone children. *Eur. J. Clin. Microbiol. Infect. Dis.* 2015;**34:**2377-2383.

29. **Zupancic K, Kriksic V, Kovacevic I, Kovacecic D.** Influence of oral probiotic *Streptococcus salivarius* K12 on ear and oral cavity health in humans: A systematic review. *Probiotics Antimicrob.* 2017. Prot. **doi: 10.1007/s12602-017-9261-2. [Epub ahead of print]:**.

30. **Horz H, Meinelt A, Houben B, Conrads G.** Distribution and persistence of probiotic Streptococcus salivarius K12 in the human oral cavity as determined by real-time quantitative polymerase chain reaction. *Oral Microbiol. Immunol.* 2007;**22:**126-130. doi: 10.1111/j.1399-302X.2007.00334.x.

31. **Levesque C, Lamothe J, Frenette M.** Coaggregation of Streptococcus salivarius with periodontopathogens: evidence for involvement of fimbriae in the interaction with Prevotella intermedia. *Oral Microbiol. Immunol.* 2003;**18:**333-337. doi: 10.1034/j.1399-302x.2003.00085.x

32. **Power DA, Burton JP, Chilcott CN, Dawes PJ, Tagg JR.** Preliminary investigations of the colonisation of upper respiratory tract tissues of infants using a paediatric formulation of the oral probiotic Streptococcus salivarius K12. *Eur. J. Clin. Microbiol. Infect. Dis.* 2008;**27:**1261-1263. doi: 10.1007/s10096-008-0569-4.

33. **Wescombe PA, Upton M, Dierksen KP, et al.** Production of the lantibiotic salivaricin A and its variants by oral streptococci and use of a specific induction assay to detect their presence in human saliva. *Appl. Environ. Microbiol.* 2006;**72:**1459-1466. doi: 72/2/1459.

34. **Di Pierro F, Colombo M, Zanvit A, Risso P, Rottoli AS.** Use of Streptococcus salivarius K12 in the prevention of streptococcal and viral pharyngotonsillitis in children. *Drug Healthc. Patient Saf.* 2014;**6:**15-20. doi: 10.2147/DHPS.S59665.

35. **Di Pierro F, Colombo M, Giuliani MG, et al.** Effect of administration of Streptococcus salivarius K12 on the occurrence of streptococcal pharyngo-tonsillitis, scarlet fever and acute otitis media in 3 years old children. *Eur. Rev. Med. Pharmacol. Sci.* 2016;**20:**4601-4606. doi: 11696.

36. **Gregori G, Righi O, Risso P, Boiardi G, Demuru G, Ferzetti A.** Reduction of group A beta-hemolytic streptococcus pharyngo-tonsillar infections associated with use of the oral probiotic Streptococcus salivarius K12: a retrospective observational study. *Ther. Clin. Risk. Manage.* 2016;**12:**87-92.

37. **Doyle H, Pierse N, Tiatia R, Williamson D, Baker M, Crane J.** Effect of oral probiotic Streptococcus salivarius K12 on Group A Streptococcus pharyngitis: a pragmatic trial in schools. *Pediatr. Infect. Dis. J.* 2018;**37:**619-623. doi: 10.1097/INF.0000000000001847.

38. **Toivonen L.** Rhinovirus infections in young children: clinical manifestations, susceptibility, and host response. PhD thesis, University of Turku, Turku; 2016.

39. **Working group set up by the Finnish Medical Society Duodecim, the Finnish Association of Otorhinolaryngology and Head and Neck Surgery, the Finnish Paediatric Society, the Finnish Association for General Practice.** 6.9.2017. Acute otitis media. *Current Care Guidelines.* **23.3.2020**.

40. **Vaz LE, Kleinman KP, Raebel MA, et al.** Recent trends in outpatient antibiotic use in children. *Pediatrics*. 2014;**133:**375-385. doi: 10.1542/peds.2013-2903.

41. **Arason VA, Kristinsson KG, Sigurdsson JA, Stefansdottir G, Molstad S, Gudmundsson S.** Do antimicrobials increase the carriage rate of penicillin resistant pneumococci in children? Cross sectional prevalence study. *BMJ*. 1996;**313:**387-391. doi: 10.1136/bmj.313.7054.387.

42. **Costelloe C, Metcalfe C, Lovering A, Mant D, Hay AD.** Effect of antibiotic prescribing in primary care on antimicrobial resistance in individual patients: systematic review and meta-analysis. *BMJ*. 2010;**340:**c2096. doi: 10.1136/bmj.c2096.

43. **Saari A, Virta LJ, Sankilampi U, Dunkel L, Saxen H.** Antibiotic exposure in infancy and risk of being overweight in the first 24 months of life. *Pediatrics*. 2015;**135:**617-626. doi: 10.1542/peds.2014-3407.

44. **Arvonen M, Virta LJ, Pokka T, Kroger L, Vahasalo P.** Repeated exposure to antibiotics in infancy: a predisposing factor for juvenile idiopathic arthritis or a sign of this group's greater susceptibility to infections? *J. Rheumatol.* 2015;**42:**521-526. doi: 10.3899/jrheum.140348.

45. **Virta L, Auvinen A, Helenius H, Huovinen P, Kolho KL.** Association of repeated exposure to antibiotics with the development of pediatric Crohn's disease--a nationwide, register-based Finnish case-control study. *Am. J. Epidemiol.* 2012;**175:**775-784. doi: 10.1093/aje/kwr400.

46. **Ortqvist AK, Lundholm C, Kieler H, et al.** Antibiotics in fetal and early life and subsequent childhood asthma: nationwide population based study with sibling analysis. *BMJ*. 2014;**349:**g6979. doi: 10.1136/bmj.g6979.

47. **Korpela K, Salonen A, Virta LJ, et al.** Intestinal microbiome is related to lifetime antibiotic use in Finnish pre-school children. *Nat. Commun.* 2016;**7:**10410. doi: 10.1038/ncomms10410.

48. **Hall MJ, Schwartzman A, Zhang J, Liu X.** Ambulatory surgery data from hospitals and ambulatory surgery centers: United States, 2010. *Natl. Health. Stat. Report.* 2017;**(102):**1-15.

49. **MRC Multi-Centre Otitis Media Study Group.** Air-conduction estimated from tympanometry (ACET) 1: relationship to measured hearing in OME. *Int. J. Pediatr. Otorhinolaryngol.* 2009;**73:**21-42. doi: 10.1016/j.ijporl.2008.09.014.

50. **Tapiainen T, Kujala T, Renko M, Koivunen P, Kontiokari T, Kristo A.** Effect of antimicrobial treatment of acute otitis media on the daily disappearance of middle ear effusion: a placebo-controlled trial. *JAMA Pediatr*. 2014;**168:**635-614.

51. **Roberts JE, Rosenfeld RM, Zeisel SA.** Otitis media and speech and language: a meta-analysis of prospective studies. *Pediatrics*. 2004;**113:**e238-48. doi: 10.1542/peds.113.3.e238.

AMENDMENTS

- The Ethical Committee of Oulu University Hospital reviewed the study protocol prior to the study on 20.4.2020 with diary decision number EETTMK 36/2020.
- The Finnish Medical Agency (FIMEA) reviewed the study prior to the study with Eudra-CT number 2020-001076-14.

Addition 28.5.2020

- The proportion of children with hospitalization due to acute respiratory illness in 0–6 months and duration of hospitalization

Amendment 8.9.2020. Due to the COVID-19 pandemic and restrictions in the day-care centers, the ear status of children will not be examined. Local child health clinics share study information pamphlets with the parents. AOM infections during the last 6 months will be inquired about via the electronic background data sheet. Filled-out informed consent forms will be gathered from day-care centers or via mail. Study physicians will deliver the study products either to day-care centers or personally. Parents are able to contact the study physicians any time via email, call, text message, or weekly open Zoom meetings.

Addition 8.9.2020. Due to the COVID-19 pandemic and restrictions at the day-care centers, the ear status of children will not be examined. MEE has been removed from the exclusion criteria.

Addition 30.9.2020. The electronic AOM diagnostic helper with 8 ear status pictures, including purulent AOM, will be sent to parents. If AOM is diagnosed, the parents will present the pictures to the attending doctor. The doctor will add the pictures that best represent the ear status to the patient records.

  - The electronic diary includes 8 different pictures of healthy tympanum, bulging and infected tympanum, and tympanum with chronic otitis media effusion. If AOM is

diagnosed, parents will present the pictures to the attending doctor, who will add the most presentative pictures to the patient records.

Amendment 30.10.2020. Due to small attendance, we expanded the recruitment area to municipal or local private day-care centers Muhos, Liminka, and Muhos and Kempele in the Oulu region, Finland.

Addition 30.10.2020

- Sachets are also suitable for children >3 years of age.

Addition 30.10.2020 We added the following outcome variables to the research protocol:

- Proportion of children with a respiratory viral sample (i.e., respiratory symptoms in 0–6 months)
- Proportion of children with COVID-19 infection or positive SARS-CoV-2 sample in 0–6 months
- Proportion of children with throat swab
- Proportion of children with *Streptococcus pyogenes* infection or positive StrepA culture in 0–6 months
- Proportion of children with other bacterial findings or viral findings in 0–6 months

Addition 30.10.2020: *Due to the COVID-19 pandemic*, the occurrence of AOM is likely to decrease due to large-scale hygiene interventions in society. We assume that the proportion of children with at least one AOM will decrease from 30% to 12% (60% relative decrease). With this new baseline occurrence, we regard 50% relative decrease as clinically significant (**12% to 6%),** with 80% power and a 5% alpha error. The required novel sample size is 356 per group (uncorrected chi-square) and 389 per group (corrected chi-square), in total 778 children. As the primary outcome will be available for all children from the comprehensive national registry, we assume a small dropout rate of 3%. Thus, we will recruit **at least 801** children.

Addition 17.12.21 We added the following outcomes to the research protocol:

- Proportion of children with at least one respiratory tract infection episode in 0–6 months
- Proportion of children who had undergone COVID-19 testing in 0–6 months
- The number of any AOM episode per child (including parent-reported AOM episodes without antibiotic prescriptions) in 0–6 months
- Proportion of children with ≥1 acute respiratory tract infection episode in 0–6 months
- Number of acute respiratory tract symptom days per child in 0–6 months
- The proportion of children with hospitalization due to acute respiratory illness in 0–6 months and duration of hospitalization for any acute reason
- Proportion of children with ≥1 parent-reported wheezing episode in 0–6 months
- Incidence of acute AOM episodes measured as verified and purchased antibiotic prescriptions in 0–6 months
- Incidence of acute AOM episodes measured as parent-reported episodes or AOM episodes that passed with watchful waiting and verified antibiotic prescriptions in 0–6 months

We removed the following secondary outcomes:

- Proportion of children with a respiratory viral sample (i.e., respiratory symptoms in 0–6 months)
- Proportion of children with throat swab
- Proportion of children with *Streptococcus pyogenes* infection or positive StrepA culture in 0–6 months
- Proportion of children with other bacterial findings or viral findings in 0–6 months
