# Supplementary material for: Streptococcus salivarius Probiotics to Prevent Acute Otitis Media in Children: A Randomized Clinical Trial
Source: JAMA Netw Open. 2023 Nov 2;6(11):e2340608. doi: 10.1001/jamanetworkopen.2023.40608 (PMC10623191; doi:10.1001/jamanetworkopen.2023.40608)
Supplement: Supplement 2. — Data Sharing Statement [file jamanetwopen-e2340608-s002.pdf]

Data Sharing Statement

Sarlin. Streptococcus salivarius Probiotics to Prevent Acute Otitis Media in Children. *JAMA Netw Open*. Published November 02, 2023. doi:10.1001/jamanetworkopen.2023.40608

Data
Data available: Yes
Data types: Deidentified participant data
How to access data: After a reasonable request for clinical research from the principal investigator, terhi.tapiainen@oulu.fi
When available: With publication

Supporting Documents
Document types: None

Additional Information
Who can access the data: After a reasonable request for clinical research from the principal investigator, terhi.tapiainen@oulu.fi
Types of analyses: For clinical research.
Mechanisms of data availability: After a reasonable request for clinical research from the principal investigator, terhi.tapiainen@oulu.fi
